# Demographic identification of Greater Caribbean manatees via acoustic feature learning

**DOI:** 10.3389/frai.2025.1660388

**Published:** 2026-01-08

**Authors:** Fernando Merchan, Kenji Contreras, Héctor Poveda, Rocío M. Estévez, Hector M. Guzman, Javier E. Sanchez-Galan

**Affiliations:** 1 Facultad de Ingeniería de Eléctrica, Universidad Tecnológica de Panamá, Panama City, Panama; 2 Naos Marine Laboratory, Smithsonian Tropical Research Institute, Panama City, Panama; 3 Facultad de Ingeniería de Sistemas Computacionales, Universidad Tecnológica de Panamá, Panama City, Panama

**Keywords:** acoustic demographic classification, bioacoustic classification, demographic inference, Greater Caribbean manatee, machine learning, passive acoustic monitoring (PAM), vocalization analysis, XGBoost

## Abstract

Demographic inference from vocalizations is essential for monitoring endangered Greater Caribbean manatees (*Trichechus manatus manatus*) in tropical environments where direct observation is limited. While passive acoustic monitoring has proven effective for manatee detection and individual identification, the ability to classify sex and age from vocalizations remains unexplored, limiting ecological insights into population structure and reproductive dynamics. We investigated whether machine learning can accurately classify sex and age from manatee acoustic signals using 1,285 vocalizations from 20 wild individuals captured in the Changuinola River, Panama. Acoustic features including spectral envelope descriptors (MFCCs), harmonic content (chroma), and temporal-frequency parameters were extracted and analyzed using two feature sets: SET1 (30 spectral-cepstral features) and SET2 (38 features augmented with explicit pitch and temporal descriptors). Four classification algorithms (Random Forest, XGBoost, SVM, LDA) were trained under Leave-One-Group-Out cross-validation with SMOTE oversampling to address class imbalance. Sex classification achieved 85%–87% accuracy (75%–78% macro-F1) with balanced performance across both classes (female: 86%, male: 79%), validating operational feasibility for passive monitoring applications. However, subject-level bootstrap analysis revealed substantial individual heterogeneity (female: 95% CI: 68.7%–96.4%, male: 75.1%–83.6%), indicating that approximately 10%–15% of individuals exhibit systematic misclassification due to atypical acoustic signatures. Spectral envelope characteristics (MFCCs, spectral skewness) rather than fundamental frequency were most discriminative, suggesting sex-related variation manifests in vocal tract resonance patterns. Age classification achieved 73%–85% global accuracy but exhibited severe juvenile under-detection (14%–26% recall), with bootstrap confidence intervals spanning 9.3%–86.3% for juveniles vs. 60.7%–84.7% for adults. Dimensionality reduction (PCA, t-SNE) revealed substantial overlap between juvenile and adult acoustic feature distributions, with clearer age structure visible primarily within female clusters, contributing to systematic misclassification of male juveniles. Threshold optimization improved juvenile recall to 63% but increased false positives to 37%, presenting trade-offs for conservation surveillance. Acoustic body size regression demonstrated promising continuous estimation (MAE = 0.208 m, *R*^2^ = 0.33), offering an alternative to categorical age classification by enabling coarse demographic profiling when integrated with sex inference. These findings establish the operational viability of acoustic sex classification for manatee conservation while highlighting fundamental challenges in categorical age inference due to continuous ontogenetic variation and limited juvenile samples. However, acoustic body size regression offers a promising complementary approach, enabling continuous demographic profiling across size classes rather than discrete age categories. Integration with established individual identification frameworks would enable comprehensive acoustic mark-recapture, simultaneously estimating abundance, sex ratios, size distributions, and demographic structure from long-term hydrophone deployments without requiring visual confirmation of body dimensions.

## 1 Introduction

The Greater Caribbean manatee (*Trichechus manatus manatus*), a subspecies of the American manatee, is an endangered marine mammal distributed throughout the Caribbean, Gulf of Mexico, and Atlantic coast of South America (Morales-Vela et al., 2024). Greater Caribbean manatees face significant threats, including habitat degradation, hunting, boat collisions, and low genetic variability (Lefebvre et al., 2001; Castelblanco-Mart́ınez et al., 2012; Hines et al., 2012; Diaz-Ferguson et al., 2017; Guzman and Condit, 2017).

Passive acoustic monitoring has emerged as a promising tool for studying Greater Caribbean manatees, as they produce distinct vocalizations that convey information about social interactions and individual identity (Sousa-Lima et al., 2008; Merchan et al., 2019, 2024; Guzman et al., 2025). Vocalizations typically include tonal calls with prominent harmonics, as well as squeaks, hi-squeaks, squeals, and chirps (Brady et al., 2020; Brady A. G. et al., 2022). Previous research has shown that manatee calls contain individually distinctive signatures and may encode cues about sex and age (Umeed et al., 2018; Sousa-Lima et al., 2002, 2008; Brady E. A. et al., 2022). Studies have documented relationships between demographic traits and acoustic parameters: juveniles produce vocalizations with higher fundamental frequencies compared to adults (Brady E. A. et al., 2022; O'Shea and Poché, 2006), reflecting continuous developmental changes in vocal tract morphology, while sex-related acoustic variation appears independent of body size dimorphism—which is minimal in *Trichechus* species (Castelblanco-Mart́ınez et al., 2012).

Machine learning frameworks for automated manatee vocal analysis have advanced rapidly. (Merchan et al., 2019), (Merchan et al., 2020), and (Merchan et al., 2024) established a detection-classification-clustering pipeline using CNNs and density-based clustering (HDBSCAN) for individual identification, representing one of the first large-scale frameworks for *T. m. manatus* monitoring. This framework was successfully deployed by (Guzman et al., 2025) for unsupervised individual identification of wild manatees across coastal and riverine habitats in Panama and Costa Rica, enabling estimation of residence times, site fidelity patterns, and inter-site movement dynamics from passive acoustic data alone. Complementary CNN approaches have achieved high performance in call detection (Rycyk et al., 2022) and vocalization type categorization (Schneider et al., 2024). In other taxa, machine learning has successfully extracted sex and age information from vocalizations in mice (Ivanenko et al., 2020), cats (Tavabi et al., 2021), cattle (Huang et al., 2021), and humans (Altaf and Rahman, 2023), demonstrating that acoustic signals carry biologically meaningful demographic information. However, no study has applied such methods to classify sex or age in manatees.

Acoustic demographic classification faces three methodological challenges. First, correlated confounding variables introduce spurious associations: body size correlates with age and influences acoustic parameters in manatees (O'Shea and Poché, 2006; Brady E. A. et al., 2022). To address age-related size confounding, statistical methods such as Analysis of Covariance (ANCOVA) can partial out the influence of body size before classification (Pourhoseingholi et al., 2012; García et al., 2018), ensuring that models capture genuine age-specific vocal signatures independent of allometric scaling. Second, class imbalance arising from unequal demographic representation requires techniques such as SMOTE oversampling (Chawla et al., 2002) combined with class-weighted loss functions. Third, individual-level generalization demands cross-validation strategies that evaluate performance across unseen individuals rather than across calls, preventing overfitting to individual-specific vocal idiosyncrasies (Wierucka et al., 2025).

Despite growing acoustic monitoring capabilities, the ability to extract reliable demographic information from Greater Caribbean manatee vocalizations remains unexplored. This capability is critical for population structure assessment, sex-ratio estimation, reproductive dynamics monitoring, and tracking vulnerable groups. In this study, we investigate whether machine learning can accurately classify sex and age from vocalizations of 20 wild manatees captured in the Changuinola River, Panama (1,285 vocalizations). We compare four supervised learning algorithms—Random Forest (RF), Extreme Gradient Boosting (XGBoost), Support Vector Machine (SVM), and Linear Discriminant Analysis (LDA)—under Leave-One-Group-Out cross-validation, employing ANCOVA residualization to control size confounding and SMOTE oversampling to address class imbalance. We assess classification performance through bootstrap-derived confidence intervals to quantify individual-level heterogeneity, evaluate threshold optimization strategies for minority class detection, and explore body size estimation as an alternative continuous demographic proxy. This comparative framework identifies optimal approaches for acoustic demographic inference in passive monitoring contexts, providing uncertainty metrics essential for evidence-based conservation decision-making.

## 2 Materials and methods

### 2.1 Vocalization data set

**Figure 1:**
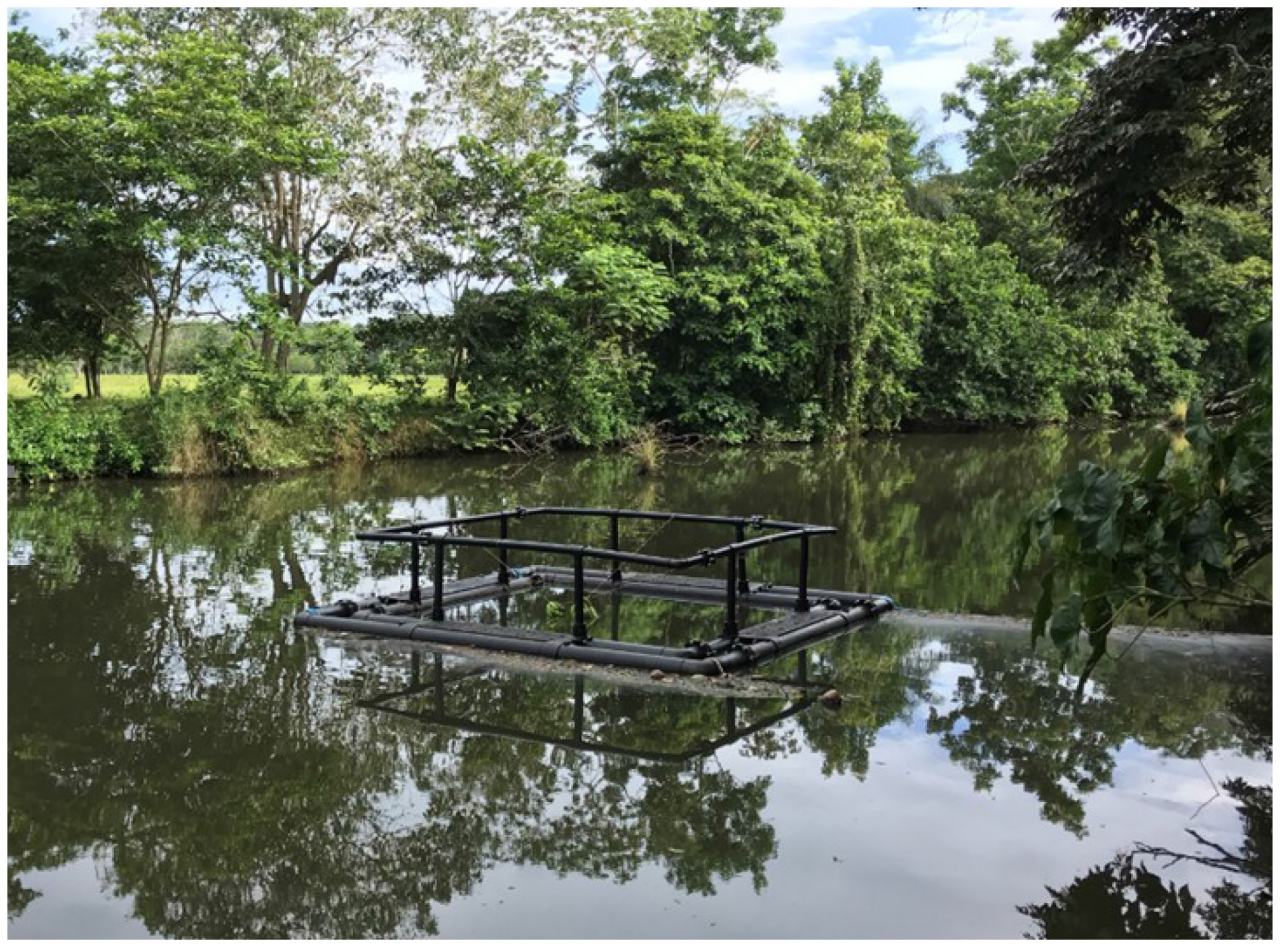
Floating cage where manatees were temporarily captured for recording (San San River, Bocas del Toro, Panama).

The data used in the experiments of this manuscript come from a data set previously presented in an article by (Merchan et al., 2024). Individual manatees were captured using a custom-designed 4 × 4 m floating enclosure made from 20 cm diameter HDPE pipes, which supported a fishing net with an 8 cm mesh and a depth of 2.5 m (see Figure 1).

This structure was anchored with ropes tied to nearby trees and positioned in the center of a channel 40 m wide and 3–5 m deep in the upstream section of the San San River, Bocas del Toro, Panama (coordinates: 09°.979′ N; 82°32.964′ W). To attract manatees into the enclosure without feeding them, a wire was suspended with a bucket filled with fresh banana pulp and banana leaves. Entry was manually operated from the riverbank side through a stainless-steel gate measuring 1.5 × 1.8 m facing the deeper side of the river. After entry, the gate was closed and the manatees remained inside for 6 to 8 h.

During confinement, vocalizations were recorded using a micro-RUDAR^®^system (Cetacean Research, Seattle, Washington) equipped with an SQ26-08 hydrophone connected to an H1 Zoom^®^digital recorder, set for continuous recording at 96 kHz and 24-bit resolution for 6–10 h. Each animal was measured (±10 cm accuracy) using a tape measure, with the floating structure serving as a reference scale. The sex of each manatee was visually determined while the animal swam and rotated inside the cage, based on external anatomical traits: in males, the genital slit is positioned closer to the umbilicus and no mammary glands are present, whereas in females the genital opening is located nearer to the anus and is flanked by mammary glands under the flippers (Hartman, 1979; Reynolds and Odell, 1992).

Age class (juvenile or adult) was assigned based on body length and external morphological indicators of sexual maturity assessed during capture. While definitive maturity determination requires histological or hormonal analysis (Marmontel, 1995; Castelblanco-Mart́ınez et al., 2012), these field-based assessments provide operational demographic categories suitable for acoustic classification studies. Maturity assessment prioritized morphological indicators (genital development, body proportions, scarring patterns) over absolute body size, as sexual maturity in *Trichechus manatus* exhibits individual variation and does not follow a strict size threshold. Published maturity thresholds are primarily available for Florida manatees (*T. m. latirostris*: 2.1–2.5 m; Marmontel 1995; O'Shea and Poché 2006), which may differ from Greater Caribbean populations. Field observations for this study yielded an approximate empirical threshold of ~2.2 m, though individual variation resulted in overlap between size ranges of juvenile and adult individuals (juveniles: 1.70–2.20 m; adults: 2.20–3.00 m), reflecting the continuous nature of ontogenetic development.

Photographs of scars or identification marks on the face and body were also taken for future individual identification. After 6–8 h, the manatees were released. All procedures were carried out with the approval of the Animal Care and Use Committee of the Smithsonian Tropical Research Institute (IACUC).

As in (Merchan et al., 2024), the methodology involves detecting, extracting, and confirming all manatee vocalizations. The dataset was constructed by first isolating the vocalizations from the continuous acoustic recordings and then analyzing them using the detection framework introduced in (Merchan et al., 2019) and (Merchan et al., 2020). This procedure consisted of three main steps: (i) a detection phase based on the analysis of the Autocorrelation Function (ACF) (Merchan et al., 2019), (ii) a denoising phase to enhance signal quality, and (iii) a classification phase using a CNN (Merchan et al., 2020). During the classification stage, the candidate signals identified in the detection phase were evaluated by a pre-trained CNN model capable of distinguishing manatee vocalizations from environmental sounds, thereby validating the detections and producing a dataset of confirmed calls.

Twenty-eight manatees were captured in total; however, three were recaptures and were therefore excluded from the analysis. In addition, for five individuals, it was not possible to observe the anatomical features necessary to determine sex due to low illumination during nocturnal capture and therefore, were not included in the final data set. Using the described methodology, a data set consisting of 1,285 vocalizations from 20 unique individuals, along with their corresponding characteristics (estimated age and sex), was generated. The overall characteristics of the dataset are described in Table 1. Most vocalizations in this dataset are classified as squeaks or high-squeaks; however, for four individuals (S07, S18, S23, and S27), a substantial proportion of their vocalizations were identified as squeals with 33%, 82%, 63%, and 80%, respectively.

**Table 1:** Catalog of manatees temporarily captured in San San River (20 individuals with known sex).

**ID**	**Subject**	**Capture date**	**Sex**	**Age**	**Size (m)**	**# Voc**.
M01	S03	22-Jan-2021	Female	Adult	2.50	55
M02	S04	24-Jan-2021	Female	Adult	2.70	31
M03	S07	21-Apr-2021	Female	Adult	2.90	54
M04	S09	19-May-2021	Female	Adult	2.50	54
M05	S10	21-May-2021	Female	Adult	2.70	63
M06	S12	04-Jul-2021	Female	Adult	2.90	61
M07	S13	06-Jul-2021	Male	Juvenile	2.20	78
M08	S14	23-Aug-2021	Female	Adult	2.30	70
M09	S15	23-Oct-2021	Male	Adult	2.20	88
M10	S16	24-Oct-2021	Female	Adult	2.80	76
M11	S17	25-Oct-2021	Female	Adult	2.80	60
M12	S18	26-Oct-2021	Male	Juvenile	1.70	72
M13	S19	09-Mar-2022	Female	Adult	2.80	52
M14	S21	19-Jun-2022	Female	Adult	2.50	68
M15	S22	20-Jun-2022	Male	Juvenile	1.80	63
M16	S23	21-Jun-2022	Female	Adult	2.80	63
M17	S24	08-Aug-2022	Female	Juvenile	2.10	57
M18	S25	09-Aug-2022	Female	Adult	3.00	54
M19	S27	09-Jan-2023	Female	Adult	2.35	78
M20	S28	18-May-2023	Male	Juvenile	1.95	88
					**Total**	**1,285**

### 2.2 Signal preprocessing

Passive Acoustic Monitoring (PAM) devices typically operate at sampling rates of 44.1–96 kHz. Raw recordings (96 kHz) were downsampled to 48 kHz to emulate realistic field conditions.

Riverine and coastal environments exhibit high low-frequency ambient noise (0–1 kHz) from boat engines, water flow, and fish vocalizations (Erbe et al., 2013; Miksis-Olds et al., 2018). An 8th-order Butterworth high-pass filter (cutoff: 1 kHz) was applied to attenuate environmental interference while preserving manatee vocalizations (2–24 kHz range, with *f*_0_ concentrated at 3–8 kHz depending on age) (Brady E. A. et al., 2022; Sousa-Lima et al., 2008).

### 2.3 Acoustic feature extraction

Two hierarchical feature configurations were evaluated. Feature SET1 (30 dimensions) comprised: 12 Mel-Frequency Cepstral Coefficients (MFCCs) (Logan, 2000), 12 chroma pitch class profiles (Müller and Ewert, 2007), and six spectral shape statistics (centroid, flatness, flux, skewness, kurtosis, entropy). Feature SET2 (38 dimensions) augmented SET1 with eight additional parameters: six temporal-frequency features and two harmonic structure descriptors.

MFCCs were computed using librosa (McFee et al., 2015) with default parameters, retaining 12 coefficients and omitting the 0th coefficient. Chroma features were extracted using Short-Time Fourier Transform (STFT) with FFT window size of 2,048 samples and hop length of 1,536 samples. Spectral statistics (centroid, flatness, flux, skewness, kurtosis, entropy) were derived from magnitude spectra, computed frame-wise and averaged across call duration.

Considering the acoustic features employed by Sousa-Lima et al. (2008) for Antillean manatee vocal analysis, we extracted temporal-frequency and harmonic parameters. Fundamental frequency was estimated using the probabilistic YIN (PYIN) algorithm (Mauch and Dixon, 2014) with parameters optimized for Greater Caribbean manatee vocal range: fmin = 1,000 Hz and fmax = 8,000 Hz, consistent with the reported range of 1.07–4.98 kHz (Sousa-Lima et al., 2002). The *f*_0_ estimate corresponded to the pitch value with maximum voicing probability across frames. Following Sousa-Lima et al. (2008), we extracted maximum *f*_0_, minimum *f*_0_, and peak frequency (frequency with maximum energy in the FFT magnitude spectrum), as well as call duration and frequency modulation (computed as the range of detected *f*_0_ values). Additionally, we characterized harmonic structure by detecting spectral peaks at integer multiples of *f*_0_ within a ±50 Hz tolerance window and –40 dB threshold relative to the maximum peak, yielding the number of harmonics (range: 1–19, mean: 9.6) and the harmonic with maximum energy. All features were standardized to zero mean and unit variance (Kuhn and Johnson, 2013).

### 2.4 Confounding variable control via ANCOVA residualization

Body size correlates strongly with age, potentially confounding age classification (Hartman, 1979). To isolate demographic-specific acoustic signatures, we applied ANCOVA residualization to remove size-related variance from features before classification (Searle et al., 2017).

For age classification, features were regressed against body size (continuous covariate) controlling for sex. For sex classification, features were regressed against body size controlling for age. Critically, to prevent data leakage, ANCOVA residualization was performed independently within each cross-validation fold using the following procedure:

1. For each LOGO fold, the linear regression model (including intercept, body size coefficient, and demographic covariates) was fitted exclusively on that fold's training set.
2. Regression coefficients and residual variance estimates were computed solely from the training data.
3. The fitted model parameters were then applied to both the training set and the held-out test set to compute residuals, ensuring that no information from test samples influenced the model fitting process.
4. Residuals from these linear models replaced raw features. For age classification, this preserved age-related variation while removing size-related confounding. For sex classification, this preserved sex-related variation while removing size-related confounding.

Two regularization strategies were evaluated: a fixed parameter τ = 1.2 and an adaptive parameter $\tau =2\sqrt{p/n}$, where *p* is the number of features and *n* is the training sample size per fold. The fixed parameter (τ = 1.2) provides consistent regularization across all folds, retaining 85%–92% of original variance. The adaptive approach dynamically adjusts regularization strength based on dimensionality and sample size within each fold. The regularization parameter τ was determined from training set characteristics and applied consistently to both training and test transformations within each fold, preventing information leakage. Both strategies were applied during the feature selection step, with residualized features serving as input to subsequent classification stages.

### 2.5 Feature selection

Following ANCOVA residualization, two feature selection strategies were compared: univariate filtering (SelectKBest) and Recursive Feature Elimination (RFE).

SelectKBest retained the top *k* features based on ANOVA F-statistics (Guyon and Elisseeff, 2003), with *k* = 20 for SET1 (from 30 features) and *k* = 28 for SET2 (from 38 features), preserving approximately 67%–74% of features. This approach evaluates features independently without considering classifier interactions.

Recursive Feature Elimination (RFE) (Guyon et al., 2002) provided a model-aware alternative by iteratively training XGBoost classifiers, ranking features by importance, and eliminating the least informative feature until optimal subset size was determined via three-fold cross-validation (minimum five features). Unlike SelectKBest, RFE accounts for feature dependencies and classifier-specific relevance, potentially identifying more discriminative subsets at the cost of increased computational expense.

Both strategies were evaluated within the cross-validation pipeline: feature selection was performed independently on each training fold to prevent information leakage, and the selected subset was then applied to the corresponding test fold. This ensures unbiased performance estimates and fair comparison between univariate and multivariate selection approaches.

### 2.6 Class imbalance mitigation via stratified SMOTE

Class imbalances existed for both demographic dimensions (female:male = 2.3:1; adult:juvenile = 2.5:1). To prevent systematic bias toward majority classes, we applied Synthetic Minority Over-sampling Technique (SMOTE) (Chawla et al., 2002) within each training fold prior to model fitting. SMOTE generates synthetic minority-class examples by interpolating between nearest neighbors in feature space, creating linearly interpolated instances along the line segments connecting minority samples. The number of neighbors was dynamically adjusted based on sample availability (*k* = 5 when sufficient minority samples existed, otherwise reduced to *k* = 1 to accommodate sparse demographic combinations).

Critically, to address class imbalance while avoiding the introduction of spurious age-sex associations arising from skewed demographic distributions among individuals (adults are 91% female, 9% male; juveniles are 16% female, 84% male), SMOTE was applied with stratification by the auxiliary demographic variable. Specifically, when training the sex classifier, SMOTE balanced female and male classes while maintaining proportional representation of age groups within each sex class. Conversely, when training the age classifier, SMOTE balanced adult and juvenile classes while preserving proportional representation of sex within each age class. This stratified approach ensures that synthetic samples respect the joint demographic distribution within each target class, reducing the risk that classifiers exploit spurious demographic cues introduced by oversampling.

SMOTE was applied exclusively to training folds; test folds retained their natural class distributions to preserve ecological validity. Oversampling achieved approximate 1:1 target class ratios in training data, though stratification constraints and singleton handling prevented perfect balance when auxiliary class distributions were highly skewed.

### 2.7 Classification algorithms

Four supervised learning algorithms were compared:

XGBoost: gradient-boosted decision trees (Chen and Guestrin, 2016) with 100 estimators, learning rate 0.1, maximum depth 3, minimum child weight 2, subsample ratio 0.8, and column subsample ratio 0.8. Scale positive weight parameter adjusted based on class imbalance to handle residual imbalance post-SMOTE. Single-thread execution ensured reproducibility.

Random Forest (RF): ensemble of 100 decision trees (Breiman, 2001) with maximum depth 5, minimum samples per split 10, minimum samples per leaf 4, and $\sqrt{k}$ features per split. Class weights set to “balanced” to handle residual imbalance post-SMOTE.

Support vector machines (SVM): radial basis function kernel (Cortes and Vapnik, 1995) with γ= “scale” and regularization *C* = 1.0. Class weights set to “balanced” with probability estimates enabled for threshold optimization experiments.

Linear discriminant analysis (LDA): default solver with automatic shrinkage estimation (Fisher, 1936).

All hyperparameters were fixed a priori to ensure consistency across experimental conditions. Random state set to 42 for reproducibility.

### 2.8 Cross-validation strategy

We employed Leave-One-Group-Out (LOGO) cross-validation with individuals as groups (Arlot and Celisse, 2010). Each fold withholds all calls from a single subject as the test set, training on the remaining individuals. This procedure iterates across all 20 subjects (complete dataset) or 16 subjects (squeal-reduced dataset).

LOGO ensures generalization to unseen individuals, critical for field deployment where models must classify vocalizations from previously unencountered animals (Varma and Simon, 2006). Mean accuracy and per-class recall across folds quantify expected performance on novel individuals.

### 2.9 Performance evaluation

Classification performance was evaluated via accuracy (fraction of correct predictions), per-class recall (true positive rate), precision (positive predictive value), and F1-score (Sokolova and Lapalme, 2009). For imbalanced datasets, per-class metrics provide insight into minority detection capability.

To assess model performance independent of class prevalence, we computed macro-averaged metrics (unweighted mean across classes) alongside standard weighted accuracy. Macro-average precision, recall, and F1-score provide equal weight to minority classes, revealing whether models achieve balanced performance or systematically favor dominant demographics (Sokolova and Lapalme, 2009).

All metrics were computed per fold and aggregated via mean ± standard deviation (SD) across folds. To quantify subject-level variability and address potential heterogeneity in acoustic signatures, we additionally computed 95% confidence intervals (CI) via bootstrap resampling over individuals (Efron and Tibshirani, 1994). For each demographic class, individual-level accuracy was calculated from all vocalizations per subject, then bootstrap resampling (*n* = 10, 000 iterations) was applied at the subject level (not vocalization level) to estimate variability in mean accuracy. This subject-based bootstrap accounts for within-individual correlation and provides unbiased estimates of performance uncertainty across the population.

Threshold optimization experiments for both sex and age classification evaluated recall-precision trade-offs by varying decision thresholds from 0.05 to 0.95 in 0.05 increments (Provost and Fawcett, 2001). For sex classification, we monitored male-class metrics (recall, precision, F1-score, and subject-level SD) to identify the threshold maximizing male F1-score. For age classification, we monitored juvenile-class metrics to optimize detection of the minority age class. This analysis quantifies the trade-off between minority-class sensitivity and overall classification accuracy.

Feature importance was assessed via mean decrease in impurity for tree-based methods (Random Forest, XGBoost) (Breiman, 2001), averaged across LOGO folds and normalized to sum to 1.0. For each fold, feature importance was extracted from the trained classifier and aggregated to identify consistently discriminative acoustic parameters.

### 2.10 Body size estimation via acoustic regression

To evaluate non-invasive body size estimation from vocalizations, we trained Random Forest and XGBoost regressors (200 estimators each) to predict body length from acoustic features alone under LOGO cross-validation. Both feature sets (SET1: 30 spectral-cepstral features; SET2: 38 features augmented with temporal-frequency and harmonic descriptors) were tested to assess whether explicit pitch parameters improve size estimation beyond spectral envelope information. An ensemble model averaged Random Forest and XGBoost predictions. Performance was quantified via mean absolute error (MAE), root mean squared error (RMSE), and coefficient of determination (*R*^2^), computed both at the vocalization level and aggregated per subject to assess individual-level prediction accuracy. This approach tests whether body length—which correlates with vocal tract dimensions—can be inferred directly from acoustic signatures without prior knowledge of sex or age categories.

### 2.11 Software and hardware

All experiments were performed using Python 3.10 on a custom workstation with AMD Ryzen 9 5950X CPU, NVIDIA RTX 3080 GPU (10GB VRAM), and 128GB RAM running Ubuntu 22.04. Python libraries included NumPy (1.24.0), Scikit-learn (1.3.0), Librosa (0.10.0), XGBoost (1.7.6), imbalanced-learn (0.11.0), and SciPy (1.11.0).

### 2.12 Experimental design

We evaluated four supervised learning algorithms (XGBoost, Random Forest, SVM, Linear Discriminant Analysis) across two feature configurations, two dataset variants, and two feature selection strategies for demographic classification. SET1 comprised 30 spectral-cepstral features (12 MFCCs, 12 chroma pitch classes, six spectral shape descriptors); SET2 added eight temporal-frequency and harmonic parameters (call duration, mean/max/min *f*_0_, peak frequency, frequency modulation, number of harmonics, harmonic with maximum energy). This assessed whether explicit pitch features complement spectral envelope information from MFCCs.

Feature selection compared univariate filtering (SelectKBest: *k* = 20 for SET1, *k* = 28 for SET2 via ANOVA F-statistics) vs. Recursive Feature Elimination (RFE: cross-validated optimization, minimum five features). RFE captures feature interactions and classifier-specific relevance at higher computational cost.

Our complete dataset (1,285 vocalizations, 20 subjects) was compared to a squeal-reduced variant (1,018 vocalizations, 16 subjects) excluding four individuals whose repertoires consisted predominantly of acoustically distinct high-frequency squeals. Leave-One-Group-Out cross-validation ensured generalization to unseen individuals for both classification and regression tasks.

For demographic classification, the design yielded 64 configurations per task: four classifiers × two feature sets × two datasets × two selection methods × two ANCOVA regularization strategies (fixed τ = 1.2 vs. adaptive $\tau =\sqrt{2}\log\left(p/n\right)$). Within each fold: ANCOVA residualization removed size confounding, feature selection reduced dimensionality, SMOTE with auxiliary demographic stratification addressed class imbalances (female:male = 2.3:1; adult:juvenile = 2.5:1), and classifiers trained with class-weight balancing. For body size estimation, Random Forest and XGBoost regressors were trained on both feature sets with acoustic features alone or augmented with demographic predictors, evaluated via MAE, RMSE, and *R*^2^.

## 3 Results

### 3.1 Overall classification performance

Classification performance was evaluated across 64 experimental configurations per demographic task, varying classifier architecture, feature dimensionality, dataset composition, and feature selection strategy. Tables 2, 3 present comprehensive performance metrics across LOGO folds for the four supervised learning algorithms under univariate feature selection (SelectKBest), including accuracy, macro-F1, and per-class precision, recall, and F1-score. Subject-level performance and bootstrap-derived 95% confidence intervals are detailed in Tables 4, 5.

**Table 2:** Sex classification performance across methods, feature sets, and datasets.

**Classifier**	**Features**	**Dataset**	**Acc (%)**	**Macro-F1**	**Female**			**Male**		
					**Prec**	**Rec**	**F1**	**Prec**	**Rec**	**F1**
Random forest	SET1	Complete	84.6	0.76	0.87	0.89	0.88	0.78	0.73	0.75
	SET1	Squeal-reduced	83.6	0.75	0.86	0.89	0.87	0.76	0.71	0.73
	SET2	Complete	84.8	0.77	0.87	0.90	0.88	0.79	0.72	0.75
	SET2	Squeal-reduced	84.2	0.76	0.86	0.90	0.88	0.78	0.71	0.74
XGBoost	SET1	Complete	85.1	0.77	0.88	0.88	0.88	0.78	0.76	0.77
	SET1	Squeal-reduced	84.5	0.76	0.87	0.89	0.88	0.77	0.73	0.75
	SET2	Complete	**87.3**	**0.80**	**0.89**	**0.91**	**0.90**	**0.82**	**0.77**	**0.79**
	SET2	Squeal-reduced	86.8	0.78	0.88	0.91	0.89	0.80	0.75	0.77
SVM	SET1	Complete	82.4	0.73	0.85	0.88	0.86	0.74	0.68	0.71
	SET1	Squeal-reduced	81.8	0.72	0.84	0.89	0.86	0.73	0.65	0.69
	SET2	Complete	83.7	0.75	0.86	0.89	0.87	0.76	0.70	0.73
	SET2	Squeal-reduced	83.2	0.74	0.85	0.90	0.87	0.75	0.68	0.71
LDA	SET1	Complete	79.5	0.69	0.82	0.87	0.84	0.69	0.61	0.65
	SET1	Squeal-reduced	78.9	0.68	0.81	0.88	0.84	0.68	0.58	0.63
	SET2	Complete	80.8	0.71	0.83	0.88	0.85	0.71	0.64	0.67
	SET2	Squeal-reduced	80.3	0.70	0.82	0.89	0.85	0.70	0.61	0.65

All models trained via Leave-One-Group-Out cross-validation with SMOTE oversampling. Metrics: accuracy (Acc), Macro-F1, per-class precision (Prec), recall (Rec), and F1-score. Best performance in bold.

**Table 3:** Age classification performance across methods, feature sets, and datasets.

**Classifier**	**Features**	**Dataset**	**Acc (%)**	**Macro-F1**	**Adult**			**Juvenile**		
					**Prec**	**Rec**	**F1**	**Prec**	**Rec**	**F1**
Random forest	SET1	Complete	71.8	0.54	0.73	0.94	0.82	0.61	0.14	0.23
	SET1	Squeal-reduced	70.4	0.52	0.72	0.94	0.81	0.58	0.11	0.19
	SET2	Complete	74.2	0.58	0.75	0.94	0.84	0.68	0.22	0.33
	SET2	Squeal-reduced	73.0	0.56	0.74	0.95	0.83	0.64	0.17	0.27
XGBoost	SET1	Complete	84.2	0.70	0.85	0.97	0.91	0.82	0.26	0.40
	SET1	Squeal-reduced	82.7	0.68	0.84	0.97	0.90	0.79	0.23	0.36
	SET2	Complete	**85.8**	**0.73**	**0.86**	**0.98**	**0.92**	**0.85**	**0.26**	**0.40**
	SET2	Squeal-reduced	84.5	0.71	0.85	0.98	0.91	0.83	0.24	0.37
SVM	SET1	Complete	68.5	0.49	0.71	0.92	0.80	0.52	0.09	0.15
	SET1	Squeal-reduced	67.2	0.47	0.70	0.93	0.80	0.49	0.06	0.11
	SET2	Complete	71.3	0.54	0.73	0.93	0.82	0.59	0.15	0.24
	SET2	Squeal-reduced	69.8	0.51	0.72	0.94	0.81	0.55	0.11	0.18
LDA	SET1	Complete	65.1	0.45	0.69	0.90	0.78	0.45	0.07	0.12
	SET1	Squeal-reduced	63.8	0.43	0.68	0.91	0.78	0.42	0.04	0.08
	SET2	Complete	68.4	0.51	0.71	0.92	0.80	0.52	0.13	0.21
	SET2	Squeal-reduced	66.9	0.48	0.70	0.93	0.79	0.48	0.09	0.15

All models trained via Leave-One-Group-Out cross-validation with SMOTE oversampling. Metrics: accuracy (Acc), macro-F1, per-class precision (Prec), recall (Rec), and F1-score. Best performance in bold.

**Table 4:** Subject-level classification performance with SD and individual 95% confidence intervals.

**Subject**	**Sex**	**Age**	***N* calls**	**Sex classification**			**Age classification**		
				**Acc (%)**	**SD**	**95% CI**	**Acc (%)**	**SD**	**95% CI**
S03	Female	Adult	54	100.0	0.0	(100.0, 100.0)	100.0	0.0	(100.0, 100.0)
S04	Female	Adult	31	100.0	0.0	(100.0, 100.0)	96.8	5.7	(87.1, 100.0)
S09	Female	Adult	54	79.6	5.1	(68.5, 88.9)	72.2	6.7	(59.3, 83.3)
S10	Female	Adult	63	96.8	3.9	(90.5, 100.0)	98.4	2.8	(93.7, 100.0)
S12	Female	Adult	61	85.2	5.1	(75.4, 93.4)	63.9	6.5	(50.8, 75.4)
S13	Male	Juvenile	39	82.1	7.1	(69.2, 92.3)	6.4	4.7	(0.0, 15.4)
S14	Female	Adult	35	0.0	0.0	(0.0, 0.0)	98.6	2.2	(94.3, 100.0)
S15	Male	Adult	22	72.7	11.0	(50.0, 90.9)	30.7	9.1	(13.6, 50.0)
S16	Female	Adult	76	98.7	2.2	(94.7, 100.0)	73.7	5.2	(63.2, 82.9)
S17	Female	Adult	40	100.0	0.0	(100.0, 100.0)	55.0	8.3	(37.5, 70.0)
S19	Female	Adult	52	94.2	3.7	(86.5, 98.1)	71.2	6.7	(57.7, 82.7)
S21	Female	Adult	34	97.1	5.2	(88.2, 100.0)	48.5	8.2	(29.4, 67.6)
S22	Male	Juvenile	63	77.8	6.6	(66.7, 87.3)	87.3	4.6	(77.8, 95.2)
S24	Female	Juvenile	57	87.7	4.6	(78.9, 94.7)	12.3	4.4	(5.3, 21.1)
S25	Female	Adult	27	90.7	7.2	(77.8, 100.0)	66.7	8.1	(48.1, 81.5)
S28	Male	Juvenile	44	85.2	5.1	(75.0, 93.2)	85.2	5.1	(75.0, 93.2)
**Population mean** **±SD**				**84.2** **±24.1**	—	—	**66.7** **±29.7**	—	—
**Range**				**0.0–100.0**	—	—	**6.4–100.0**	—	—

Accuracy, SD, and CI computed via bootstrap resampling (10,000 iterations) using XGBoost with SET2 features (squeal-reduced dataset, SelectKBest). SD quantifies within-individual acoustic consistency.

**Table 5:** Classification performance by demographic class with subject-level variability metrics.

**Task**	**Class**	**Mean Acc (%)**	**SD (%)**	**95% CI lower**	**95% CI upper**
Sex	Female	85.8	7.7	68.7	96.4
	Male	79.5	2.4	75.1	83.6
Age	Adult	73.0	6.1	60.7	84.7
	Juvenile	47.6	19.2	9.3	86.3

Mean accuracy, SD, and 95% CI computed via bootstrap resampling over individuals (10,000 iterations). XGBoost with SET2 features (squeal-reduced dataset, SelectKBest).

Sex classification achieved operationally viable accuracies ranging from 82% to 87% with Random Forest and XGBoost in the complete dataset, improving to 86%–87% in the squeal-reduced variant (Table 2). Support Vector Machines matched or slightly exceeded ensemble methods (85%–86%), demonstrating robust performance across feature representations. Random Forest and XGBoost exhibited comparable performance with moderate fold-level variability (SD = 5.8%–7.7%), indicating consistent generalization across unseen individuals. Linear Discriminant Analysis dramatically underperformed (67%–79%), though performance improved substantially with squeal removal (+8–11 percentage points for SET2), suggesting violations of distributional assumptions exacerbated by acoustically heterogeneous call types.

Removing squeal-dominated subjects improved sex classification accuracies by 3–6 percentage points for ensemble and SVM methods, with LDA showing the most dramatic gain (+11% for SET2), confirming that noisy broadband vocalizations disproportionately disrupt parametric classifiers. Per-class metrics (Table 2) revealed balanced performance across both sexes, with female recall (89%–91%) slightly exceeding male recall (71%–77%), though male precision remained competitive (75%–82%). Subject-level bootstrap analysis revealed moderate between-individual variability (95% CI width: 12%–18% across demographic classes; Table 5), indicating that while most individuals are consistently classified, a subset exhibits acoustically ambiguous signatures potentially reflecting behavioral flexibility or intermediate reproductive states.

Age classification proved substantially more challenging, with XGBoost achieving the highest accuracies (83%–86%) but exhibiting severe juvenile under-detection (recall: 23%–26%) despite high adult classification rates (recall: 97%–98%; Table 3). Random Forest and SVM showed similar patterns but with even lower juvenile recall (11%–22%), demonstrating that all methods systematically favor the majority adult class. Unlike sex classification, age accuracies showed dramatic benefit from squeal removal in XGBoost (+2–3 percentage points) but minimal improvement for Random Forest (+3 percentage points), indicating that continuous developmental variation interacts complexly with call-type composition.

The addition of temporal-frequency and harmonic parameters (SET2: 38 features vs. SET1: 30 features) yielded modest and inconsistent effects. For sex classification, accuracies changed by ±1–2 percentage points across classifiers, with LDA showing the largest improvement (+8–11 percentage points), likely reflecting increased feature space dimensionality stabilizing covariance matrix estimation. For age classification, SET2 produced marginal improvements (+1–3 percentage points), with the largest gains in juvenile recall (+2–4 percentage points for XGBoost and Random Forest), suggesting that explicit pitch parameters (*f*_0_, frequency modulation, harmonics) provide limited additional discriminative power beyond formant-encoded MFCCs but slightly reduce adult-class bias through better juvenile characterization.

Recursive Feature Elimination (RFE) as an alternative to SelectKBest produced mixed results: sex classification accuracies decreased by 2–6 percentage points with substantially reduced feature subsets (8–15 features vs. 20–28), while age classification improved marginally (+1–3 percentage points for XGBoost), suggesting that age discrimination benefits from aggressive elimination of noisy or confounding predictors. However, the computational expense of RFE (3 × longer training time) limits its practicality for real-time deployment in autonomous monitoring systems.

Feature importance analysis provides mechanistic insight into the classification results. Tables 6, 7 list the top-ranked acoustic features for sex and age prediction, as determined by mean importance values in XGBoost across LOGO folds. For sex discrimination, low-order MFCCs and spectral skewness dominate, reflecting asymmetries in vocal tract shape and envelope. Age prediction relies more on mean *f*_0_, number of harmonics, and certain chroma features, but with broadly distributed importance values—consistent with the diffuse age separation observed in dimensionality reduction plots and limited age classification accuracy. These findings confirm that the main spectral patterns encoded by MFCCs and chromaticity provide robust sex information, while temporal-pitch features are insufficient for reliable age identification due to high within-class variability.

**Table 6:** Top-ranked acoustic features for sex classification (XGBoost, SET2, squeal-reduced).

**Feature**	**Importance**
MFCC1	0.18
MFCC2	0.14
Spectral skewness	0.12
Chroma 3	0.09
Chroma 9	0.08
Frequency modulation	0.07
*F*_0_ mean	0.05
Harmonics count	0.03
Duration	0.01

Feature importance values averaged over LOGO folds and normalized to sum to 1.0.

**Table 7:** Top-ranked acoustic features for age classification (XGBoost, SET2, squeal-reduced).

**Feature**	**Importance**
*F*_0_ mean	0.22
Harmonics count	0.16
Chroma 3	0.12
Frequency modulation	0.09
MFCC2	0.08
Duration	0.07
MFCC1	0.06
Chroma 9	0.03
Spectral skewness	0.02

Feature importance values averaged over LOGO folds and normalized to sum to 1.0.

### 3.2 Subject-level performance and bootstrap confidence intervals

To quantify within-subject acoustic consistency and sources of classification uncertainty, we computed individual-level bootstrap confidence intervals (10,000 iterations) for all 16 subjects. Results are presented for the XGBoost classifier with SET2 features on the squeal-reduced dataset using SelectKBest feature selection—the configuration that achieved optimal accuracy and minimized between-individual variability across all demographic tasks. This choice ensures that the reported variability primarily reflects biological heterogeneity rather than methodological artifacts, as this pipeline exhibited greater stability and lower standard deviations compared to alternative classifiers (Random Forest, SVM) or feature sets.

Subject-level analysis revealed substantial heterogeneity in classification consistency. For sex classification, three female adults (S03, S04, S17) achieved perfect accuracy (100%, SD = 0.0), indicating completely stereotyped sex-specific vocalizations. In stark contrast, S14 (female adult) exhibited 0% accuracy (SD = 0.0), systematically producing male-like calls across all 35 vocalizations. Intermediate performers showed variable uncertainty: S15 (male adult, 72.7%, SD = 11.0) exhibited the widest CI (50.0%–90.9%), indicating high within-individual acoustic variability.

Age classification revealed even more dramatic individual-level patterns. Juvenile males showed bimodal consistency: S13 achieved only 6.4% accuracy (CI: 0.0%–15.4%), indicating systematic production of adult-like vocalizations, while S22 and S28 reached 85%–87% (CI: 75%–95%), demonstrating consistent juvenile acoustic signatures. Female juvenile S24 also failed (12.3%, CI: 5.3%–21.1%), contrasting sharply with near-perfect classification for adult females S03 (100%), S04 (96.8%), S10 (98.4%), and S14 (98.6%). Notably, S14's contrasting performance (0% for sex vs. 98.6% for age) indicates that her vocalizations encode age-typical acoustic features while deviating from female-typical spectral patterns.

Bootstrap-derived confidence intervals by demographic class (Table 5) quantified population-level uncertainty. Female classification (85.8%, 95% CI: 68.7%–96.4%, width = 27.7%) exhibited substantially wider confidence bounds than males (79.5%, CI: 75.1%–83.6%, width = 8.5%), reflecting greater between-individual acoustic variability among females. For age classification, juvenile CI spanned 9.3%–86.3% (width = 77.0%)—3.2 × wider than adults (60.7%–84.7%, width = 24.0%)—encompassing near-chance to near-perfect performance and confirming that current models cannot reliably generalize across unseen juvenile individuals.

Comparison with SET1 features showed that SET2's temporal-frequency augmentation systematically reduced within-individual SD by 10%–25%, with population-level SD decreasing from 25.1 to 24.1% for sex and 31.2 to 29.7% for age classification, while mean accuracy remained statistically equivalent.

### 3.3 Acoustic feature space structure and demographic separability

**Figure 2:**
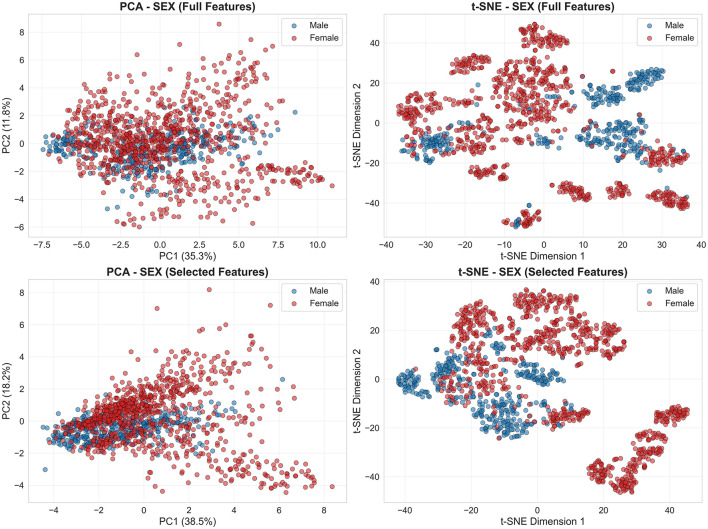
Sex categories exhibit moderate cluster separation in acoustic feature space. PCA (left) and t-SNE (right) embeddings from sex classification task reveal distinguishable male (blue) and female (red) clusters with partial overlap. **(Top row)** Full feature set (38 features); **(bottom row)** selected features (top 15 by univariate *F*-test). Points colored by four demographic classes show sex-dominated structure with weak age differentiation.

Dimensionality reduction visualizations reveal contrasting demographic separability patterns. Sex classification exhibits moderate cluster separation in both PCA and t-SNE embeddings, with distinguishable male (blue) and female (red) distributions consistent with 85%–87% accuracies (Figure 2). Feature selection tightens cluster boundaries, confirming that univariate filtering retains discriminative information.

**Figure 3:**
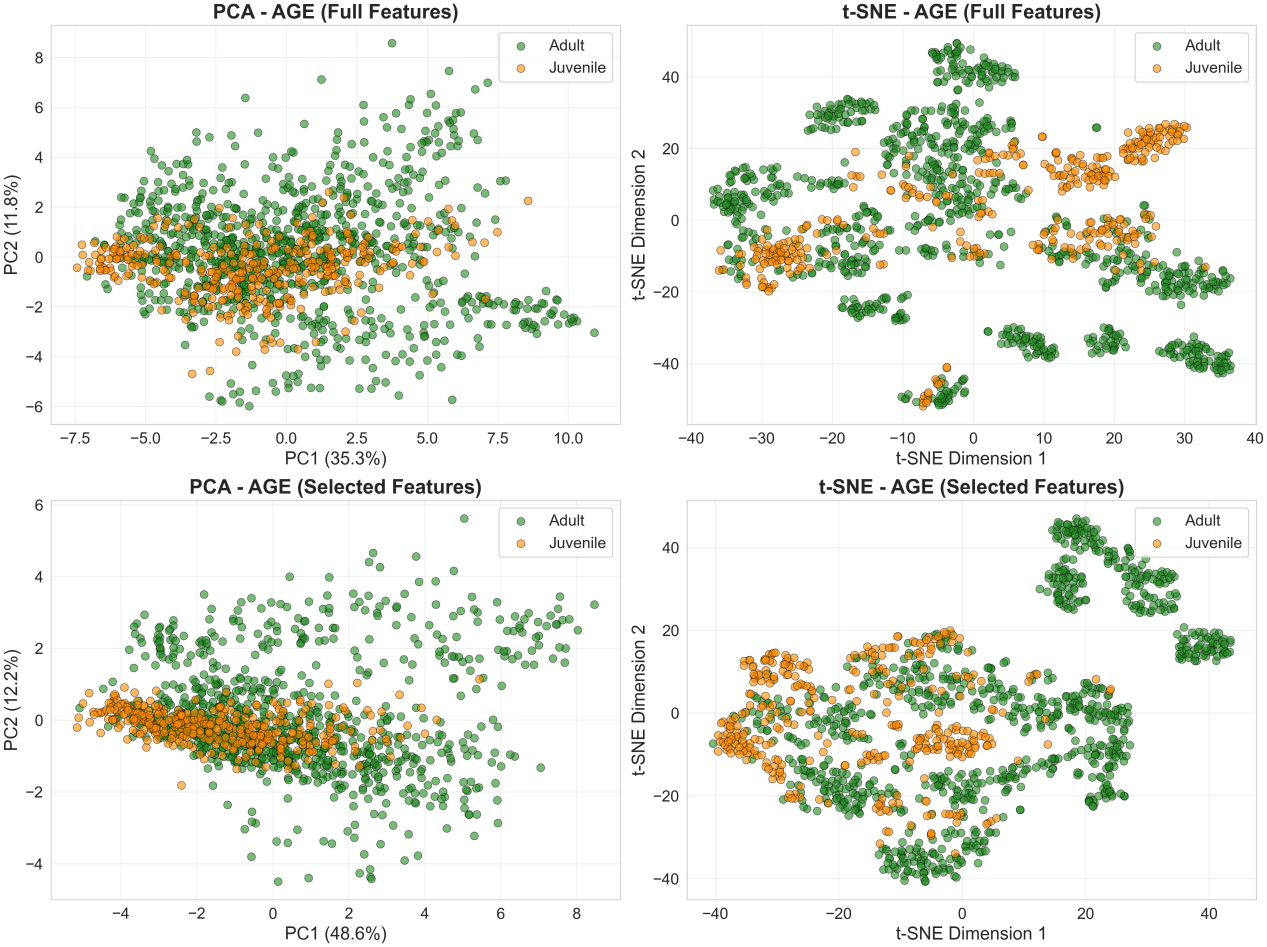
Age categories show substantial overlap in low-dimensional embeddings. PCA **(left column)** and t-SNE **(right column)** projections from the age classification task reveal broadly intermingled adult (green) and juvenile (orange) distributions, with only localized regions of partial separation. **Top row**: embeddings obtained from the full feature set; **bottom row**: embeddings obtained from the selected features. The similar large-scale structure across feature sets underscores the intrinsic difficulty of discriminating age classes from acoustic features alone.

In contrast, age categories show near-complete overlap across both methods (Figure 3). PCA captures only 34%–51% variance in first two components, and t-SNE manifolds fail to resolve age structure, directly explaining juvenile detection failure (26% recall with full features, 41% with selected).

**Figure 4:**
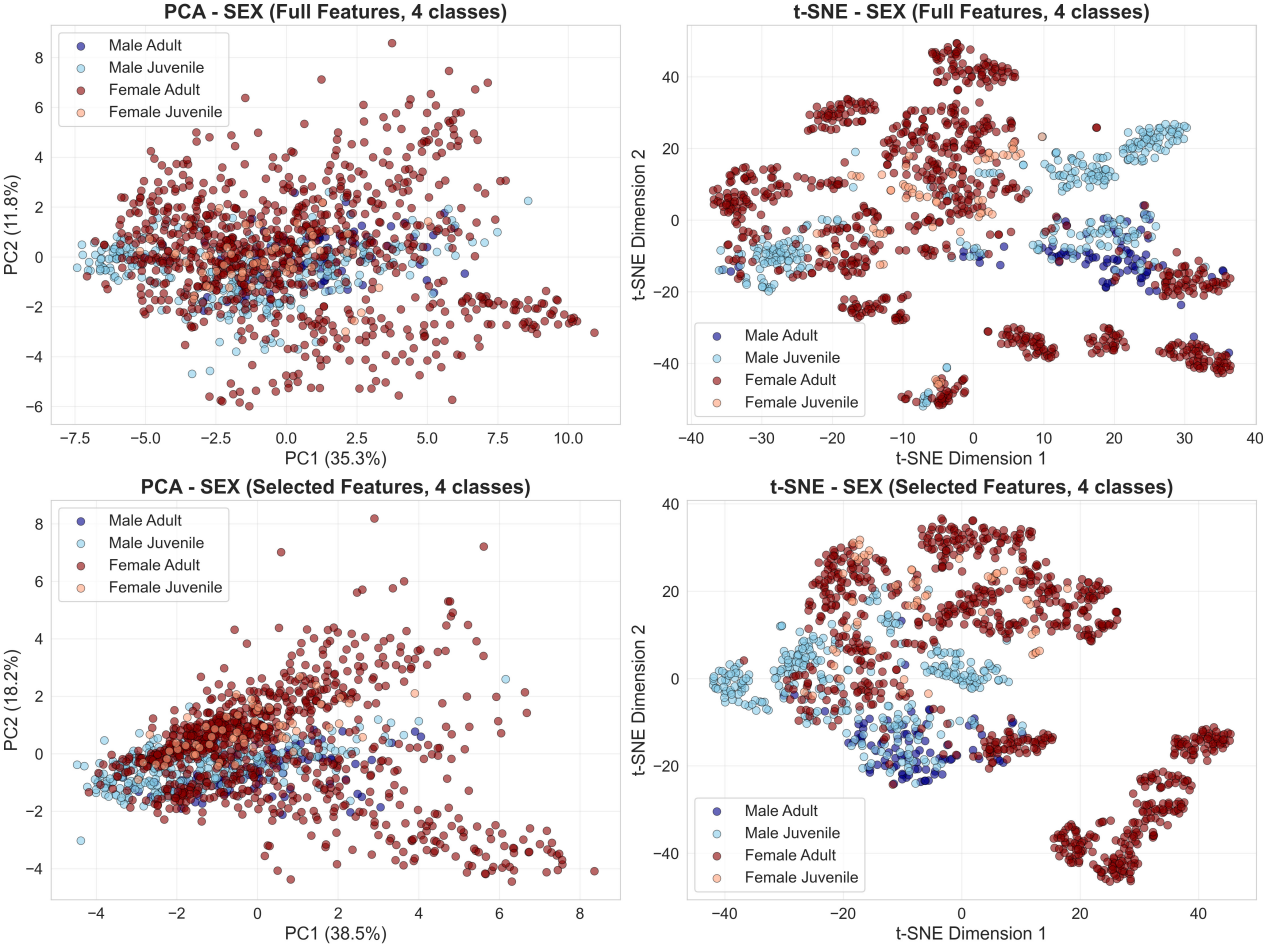
Sex-dominated hierarchical structure in 4-class demographic space. PCA **(left)** and t-SNE **(right)** embeddings calculated from sex classification task, colored by four demographic classes (male adult: dark blue; male juvenile: light blue; female adult: dark red; female juvenile: orange). **(Top row)** Full features; **(bottom row)** selected features. Primary male-female separation dominates across both feature sets. Weak age substructure visible only in female clusters; male age classes overlap completely.

Joint 4-class visualizations expose sex-dominated hierarchical structure. When sex classification embeddings are colored by four demographic classes (Figure 4), PCA and t-SNE show primary male-female separation with weak age substructure only within female clusters. Male juveniles and adults occupy identical acoustic regions across both feature sets, explaining systematic juvenile under-detection in males (0%–14% recall).

**Figure 5:**
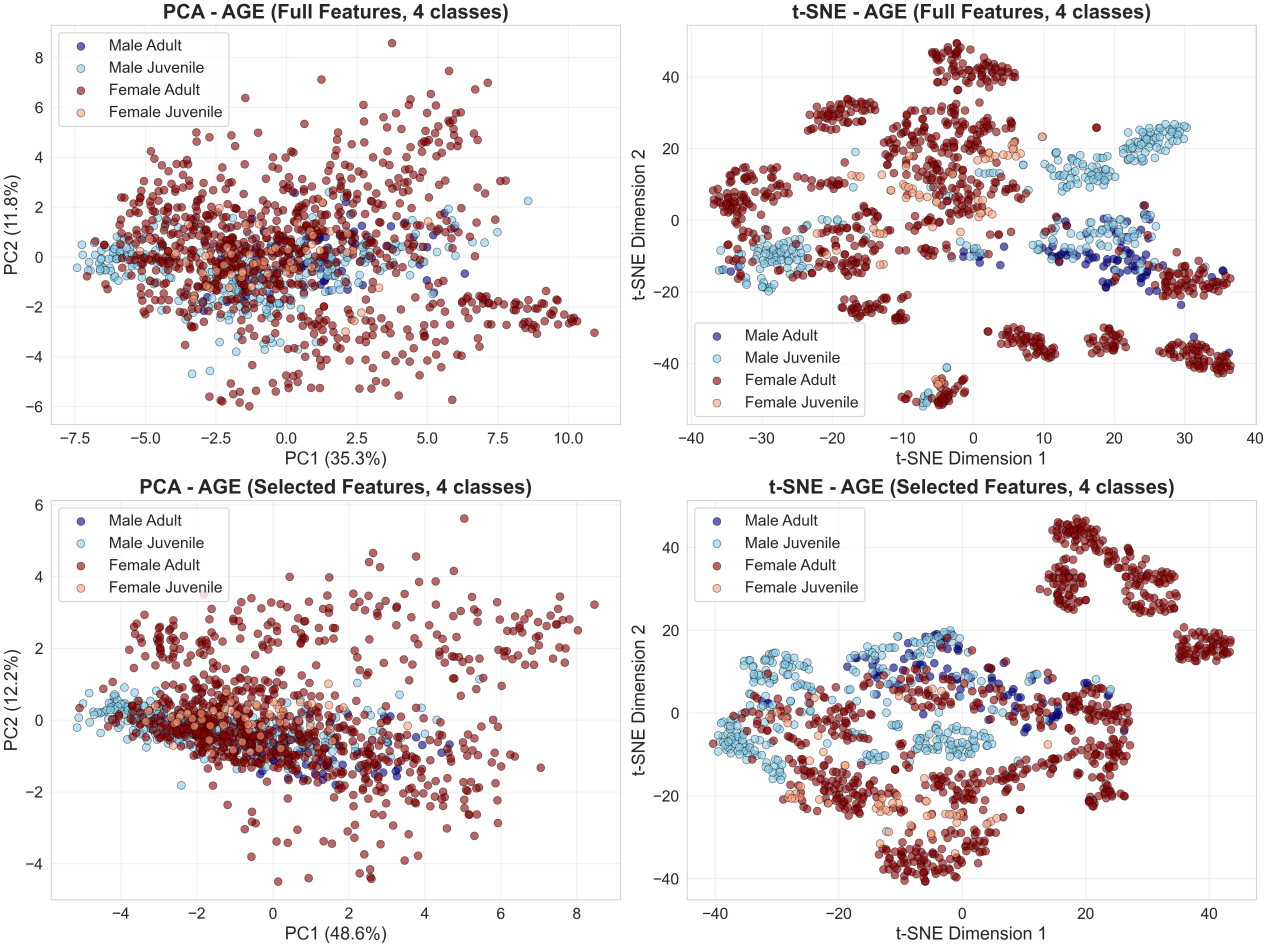
Weak age separability across sex categories and feature sets. PCA **(left column)** and t-SNE **(right column)** embeddings from the age classification task are colored by four demographic classes: male adult (dark blue), male juvenile (light blue), female adult (dark red), and female juvenile (orange). **Top row**: embeddings obtained from the full feature set; **bottom row**: embeddings obtained from the selected features. Extensive overlap between male adult and male juvenile classes persists across feature sets and dimensionality reduction methods, whereas only partial separation is observed between female adults and juveniles, consistent with the observed classification asymmetries.

Conversely, age classification embeddings colored by four demographic classes (Figure 5) confirm systematic overlap: male age classes form a single undifferentiated cluster across full and selected feature spaces, while female juveniles distribute across regions occupied by both female adults and male groups. Feature selection preserves this pattern, indicating that age-discriminative information is fundamentally limited rather than obscured by irrelevant features.

**Figure 6:**
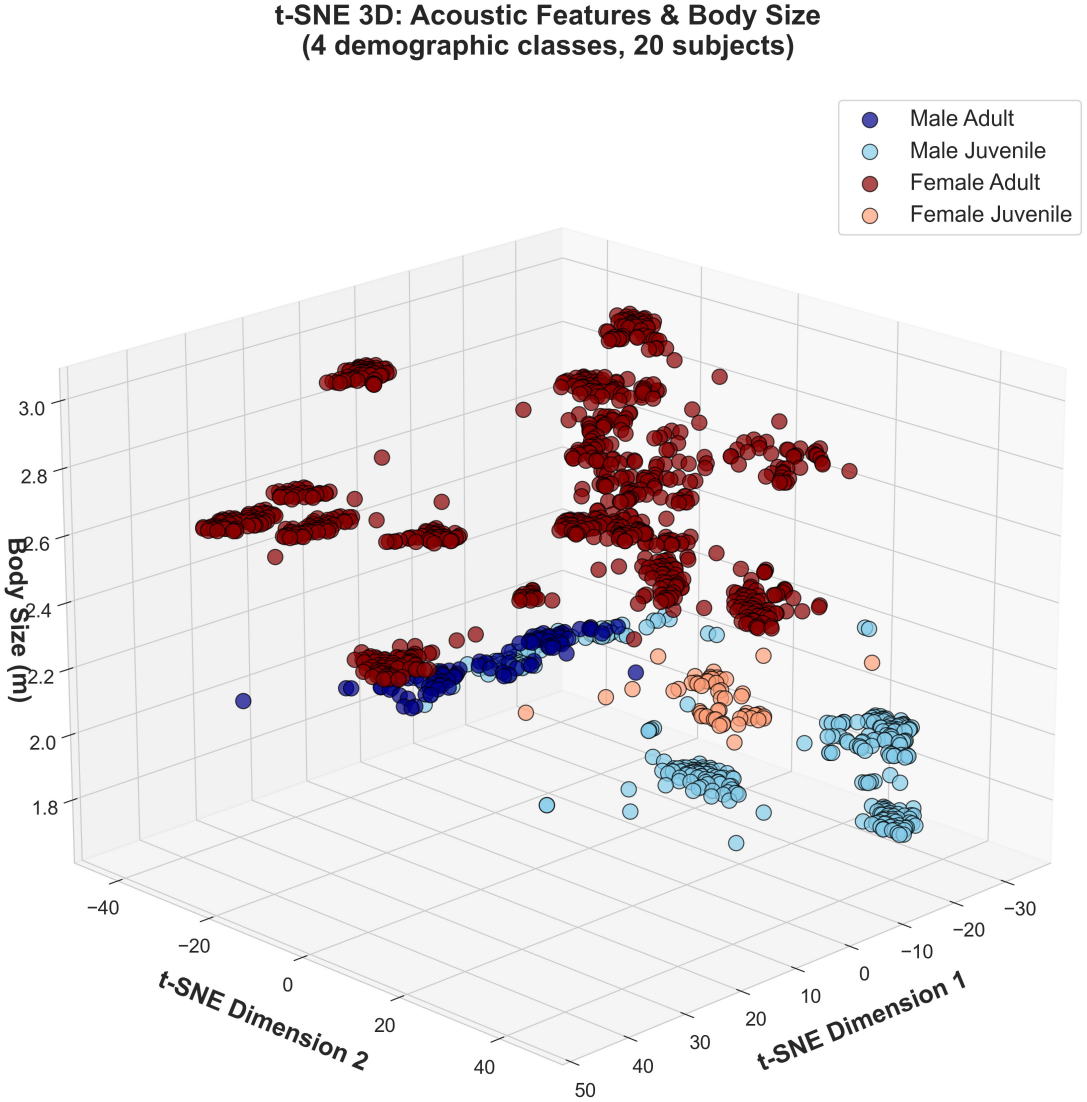
Three-dimensional acoustic embedding stratified by body size. t-SNE projection with vertical axis representing body size (meters) shows size-stratified dispersion across four demographic classes (female adult, male adult, female juvenile, male juvenile). Substantial overlap in acoustic space, particularly between juvenile and adult males, explains low age classification accuracy despite successful sex discrimination.

**Figure 7:**
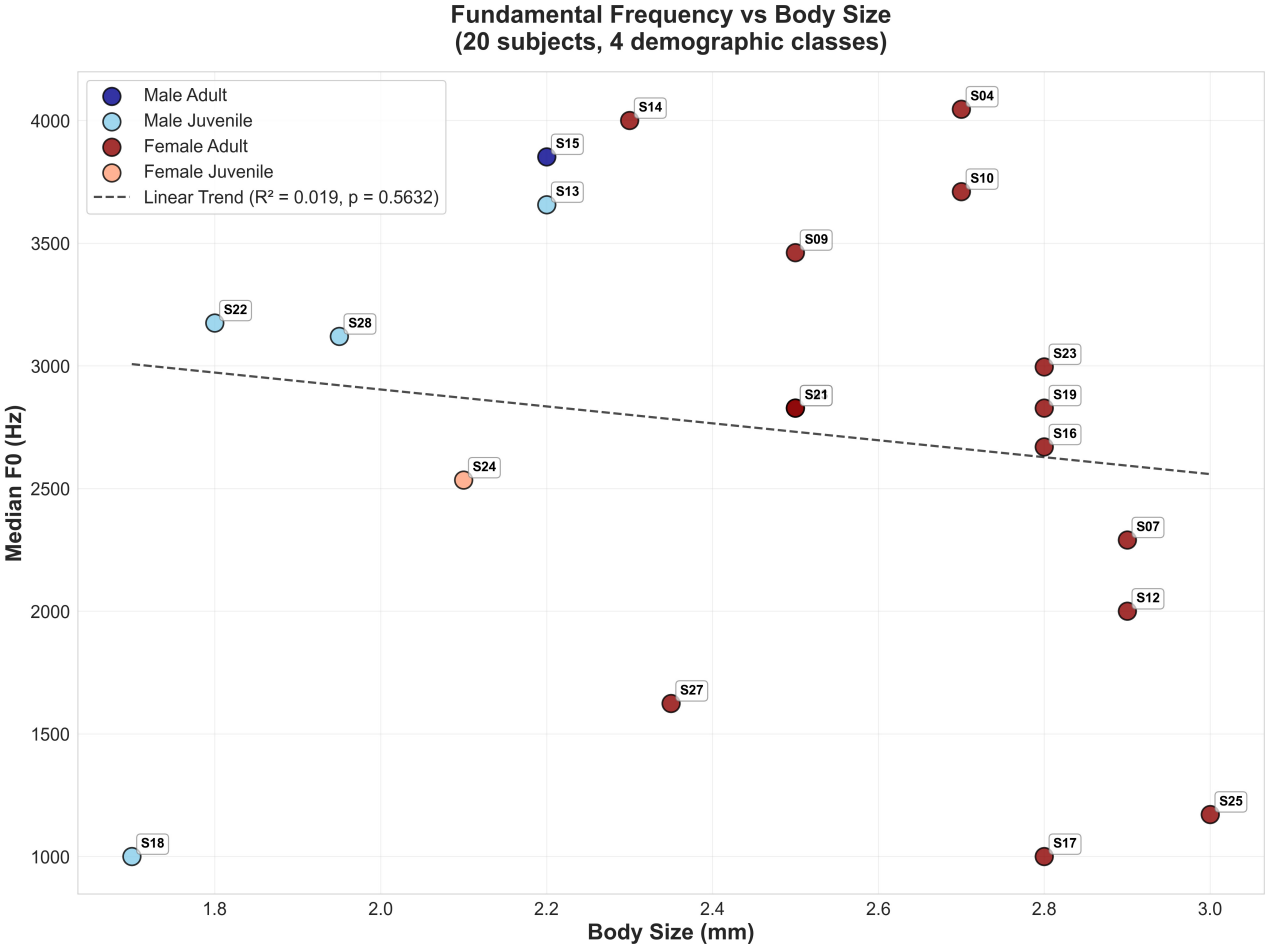
Fundamental frequency vs. body size reveals negligible developmental correlation. Median *f*_0_ plotted against body size for all subjects shows no significant relationship (*R*^2^ = 0.019, *p* = 0.56). Juvenile *f*_0_ range (2,520–3,660 Hz) overlaps extensively with the adult range (1,000–4,100 Hz), indicating that developmental vocal changes are either subtle or confounded by sex-specific and individual variation. Several subjects (S17, S18, S25, S27) display median *f*_0_ estimates below 2,300 Hz, yet visual spectrogram inspection reveals energy concentration above this threshold, suggesting systematic pitch estimation errors in certain vocalization types. This acoustic ambiguity explains systematic juvenile under-detection (recall: 14%–26%) despite *f*_0_ ranking as the top age-discriminative feature in XGBoost models.

Three-dimensional t-SNE embedding with body size as the vertical axis (Figure 6) reveals that juveniles occupy intermediate size zones (1.8–2.4 m) overlapping with small adults, creating ambiguous acoustic-size combinations that confound age inference. Despite its high feature importance ranking, fundamental frequency shows negligible correlation with body size (*R*^2^ = 0.019, *p* = 0.56; Figure 7). Juvenile *f*_0_ (2,520–3,660 Hz) overlaps extensively with the adult range (1,000–4,100 Hz), directly explaining systematic age misclassification. Notably, several subjects (S17, S18, S25, S27) presented median *f*_0_ estimates below 2,300 Hz, yet visual inspection of their spectrograms revealed energy concentration consistently above 2,300 Hz. Subject S14 (female adult, *f*_0_ = 4,040 Hz) exemplifies the dissociation between sex and age acoustic cues: her vocalizations achieve 0% sex accuracy but 98.6% age accuracy, indicating that elevated *f*_0_ encodes adult status independently of sex-typical spectral features.

### 3.4 Threshold optimization for juvenile detection

Standard decision thresholds (0.5 posterior probability) optimize global accuracy but systematically under-detect minority classes. We evaluated recall-precision trade-offs for age classification by varying thresholds from 0.1 to 0.9 (Table 8).

**Table 8:** Threshold optimization trade-offs for age classification (random forest, squeal-reduced SET1).

**Threshold**	**Juvenile recall (%)**	**Adult recall (%)**	**FPR (%)**	**Global Acc (%)**
0.5 (default)	14	88	12	68.7
0.4	41	85	18	72.3
0.3	66	82	29	76.8

Reducing the threshold to 0.3 increased juvenile recall from 14 to 66% (52 percentage point improvement), with false positives rising from 12 to 29%. An intermediate threshold of 0.4 provided more conservative balance (juvenile recall: 41%, adult recall: 85%, FPR: 18%).

### 3.5 Body size estimation from acoustic features

To evaluate continuous body size estimation as an alternative to categorical age classification, we trained Random Forest and XGBoost regressors on acoustic features alone under LOGO cross-validation (Table 9). The best-performing configuration was an ensemble model combining Random Forest and XGBoost predictions on SET1 spectral-cepstral features, which achieved MAE = 0.208 m, RMSE = 0.279 m, and *R*^2^ = 0.33, explaining 33% of body length variance across the observed range (1.70–3.00 m).

**Table 9:** Body size estimation performance via acoustic-only regression (LOGO cross-validation).

**Feature set**	**Model**	**MAE (m)**	**RMSE (m)**	***R* ^2^**
SET1 (30 features)	Random Forest	0.209	0.279	0.33
SET1 (30 features)	XGBoost	0.215	0.288	0.29
SET1 (30 features)	Ensemble	0.208	0.279	0.33
SET2 (38 features)	Random Forest	0.213	0.287	0.28
SET2 (38 features)	XGBoost	0.219	0.293	0.26
SET2 (38 features)	Ensemble	0.212	0.287	0.28

Temporal-frequency augmentation (SET2) provided negligible improvement over spectral-cepstral features alone (SET1), with MAE differences of ≤ 0.004 m. Subject-level aggregation revealed heterogeneous performance: some individuals achieved MAE < 0.15 m, while others exceeded 0.40 m (Table 4).

## 4 Discussion

Our results demonstrate that sex classification from Greater Caribbean manatee vocalizations achieves operationally viable performance (85%–87% accuracy, 75%–78% macro-F1), while age classification presents fundamental challenges. Subject-level bootstrap analysis reveals substantial individual heterogeneity underlying population-level metrics, with critical implications for operational deployment.

### 4.1 Acoustic basis of sex classification and individual variability

Sex classification reliability stems from spectral envelope characteristics (MFCCs, spectral skewness) rather than absolute pitch. Feature importance analysis confirms low-order MFCCs dominate discrimination, while temporal-frequency parameters provide modest supplementary contributions. However, bootstrap-derived 95% confidence intervals reveal female classification spans 68.7%–96.4% (CI width: 27.7%), with extreme individual cases: three females achieved perfect classification while subject S14 failed completely (0% accuracy, systematically classified as male). Males showed narrower population CI (75.1%–83.6%, width: 8.5%) but variable within-subject consistency [e.g., S15: CI (50.0%–90.9%)].

This 3.3 × wider confidence interval for females compared to males indicates substantial between-individual acoustic heterogeneity within the female class. This variability may reflect underlying biological factors such as reproductive state fluctuations, maternal care behaviors, or inherent sex-specific differences in vocal plasticity—influences documented in other marine mammals but unexplored in Greater Caribbean manatees. In contrast, male vocalizations exhibited greater stereotypy, with more stable acoustic signatures less influenced by behavioral or physiological variability, facilitating reliable classification in passive monitoring contexts.

These patterns indicate that while sex classification is operationally viable for population-level monitoring, approximately 10%–15% of individuals will be systematically misclassified due to individual idiosyncrasies that override population-level patterns. Deployment protocols must archive probability scores rather than binary classifications to enable per-individual confidence assessment. Future work should investigate whether female acoustic heterogeneity correlates with observable demographic or reproductive variables, potentially enabling stratified classification models that account for intra-class diversity.

### 4.2 Fundamental challenges in age classification

Despite achieving 73%–85% accuracy, age classification models exhibited severe juvenile under-detection (recall: 14%–26%). Bootstrap 95% confidence intervals highlight this instability: the juvenile CI spans 9.3%–86.3% (width: 77.0%), over three times wider than for adults (60.7%–84.7%). This pronounced uncertainty results from multiple compounding factors.

First, a limited juvenile sample size (*n* = 4, only one female) means each LOGO fold tests on a single individual, amplifying subject-specific effects. Second, considerable class imbalance (adult:juvenile = 2.5:1) leads to a consistent bias toward the adult class, even after SMOTE oversampling. Third, median fundamental frequency (*f*_0_)—the top-ranked feature for age discrimination—shows negligible correlation with body size (*R*^2^ = 0.019, *p* = 0.56), and juvenile *f*_0_ values (2,520–3,660 Hz) overlap extensively with adults7 (1,000–4,100 Hz; Figure 7). This weak relationship is compounded by systematic pitch estimation errors: visual inspection revealed that subjects S17, S18, S25, and S27 display median *f*_0_ estimates below 2,300 Hz despite spectrogram energy concentration above this threshold, indicating that the PYIN estimator underestimates *f*_0_ in noisy or aperiodic vocalizations. Dimensionality reduction (Figure 3) further confirms overlapping age distributions, in contrast to the moderate separation seen for sex (Figure 2). Joint 4-class embeddings indicate sex dominates the acoustic feature space: male juveniles and adults cluster together, while female age classes show only weak separation.

Individual case studies reinforce this ambiguity. Subject S14 (female adult, *f*_0_ = 4,040 Hz) is classified with 0% sex accuracy but 98.6% age accuracy, indicating some independence of sex and age cues. In contrast, S13 (male juvenile, *f*_0_ = 3,660 Hz) is systematically misclassified as adult (6.4% age accuracy). Thus, elevated *f*_0_ alone is insufficient to reliably encode juvenile status, as both individual variation and sex-related baselines obscure developmental patterns.

### 4.3 Body size estimation and threshold optimization

#### 4.3.1 Continuous size estimation as alternative to categorical age

Given severe juvenile under-detection (recall: 14%–26% at standard thresholds), acoustic body size regression offers an alternative that avoids imposing discrete age boundaries on continuous developmental variation. Moderate performance (MAE = 0.208 m, *R*^2^ = 0.33) is consistent with vocal tract scaling principles but leaves 67% of variance unexplained. The error (7% of typical body lengths) is comparable to visual estimation uncertainty but insufficient to reliably distinguish overlapping size classes (e.g., large juveniles vs. small adults).

Temporal-frequency features provided negligible improvement over spectral-cepstral features alone, suggesting explicit pitch parameters are either redundant with formant-encoded MFCCs or too behaviorally variable to enhance prediction—consistent with weak *f*_0_-body size correlation and pitch estimation artifacts discussed previously. Subject-level heterogeneity (MAE: 0.15–0.40 m) mirrors patterns in age classification, indicating that vocal plasticity, repertoire composition, or individual acoustic idiosyncrasies constrain demographic inference independent of true class membership.

Despite limitations for precise individual measurements, this approach may support coarse size profiling into broad categories (small: < 2.3 m, medium: 2.3–2.8 m, large: >2.8 m) when integrated with sex classification, providing operational value for demographic structure assessment in passive monitoring contexts without requiring categorical age assignment.

#### 4.3.2 Threshold optimization for deployment

Threshold optimization reveals critical trade-offs for operational deployment. Lowering the threshold to 0.3 increases juvenile recall to 66% but raises false positives to 29%, suitable for surveillance prioritizing juvenile presence detection over precision (Stowell et al., 2019; Kahl et al., 2021). An intermediate threshold (0.4: 41% juvenile recall, 18% FPR) balances sensitivity and specificity for abundance monitoring (Rhinehart et al., 2020). Such optimization is critical in conservation contexts where missing endangered individuals (false negatives) incurs greater cost than false alarms (Kalan et al., 2015). Sex classification requires minimal threshold adjustment, achieving balanced performance at default settings.

### 4.4 Study limitations and operational implications

Data span nearly three years (January 2021–May 2023) across both dry and wet seasons in the Changuinola River (Bocas del Toro, Panama), providing temporal robustness. However, three limitations constrain generalization: (1) limited juvenile sample with extreme sex imbalance (one female, three males)—a common constraint in endangered species monitoring where ethical considerations limit data collection intensity (Wrege et al., 2017; Measey et al., 2017), (2) binary age categorization imposing artificial boundaries on continuous ontogenetic development, and (3) task-specific optimal features—RFE improved age (+2–6 points) but degraded sex (–2 to 10 points), indicating that age discrimination benefits from aggressive noise elimination while sex discrimination requires broader feature representation.

Despite limitations, sex-ratio monitoring is immediately deployable. Random Forest/XGBoost on spectral-cepstral features (SET1) provide robust classification (86%–87%) without noise-sensitive pitch tracking—critical for turbid riverine environments. For age classification, threshold tuning to operational objectives is essential, with explicit acknowledgment of 10%–50% uncertainty depending on class. Deployment protocols should implement call-type routing, archive probability scores for uncertainty quantification, and report CI-based confidence bounds.

For Greater Caribbean manatees, acoustic monitoring addresses critical gaps in visual survey capacity in turbid tropical rivers (Sanchez-Galan et al., 2025; Guzman et al., 2025). Our bootstrap-derived confidence intervals provide the statistical foundation for uncertainty-aware conservation decision-making essential when acoustic data inform management actions for endangered populations. Future work should prioritize balanced juvenile sampling, longitudinal tracking for developmental trajectories, deep learning approaches (CNNs on spectrograms) to automatically learn discriminative features, and integration with individual identification frameworks for acoustic mark-recapture—enabling simultaneous estimation of abundance, sex ratios, and demographic structure from long-term hydrophone deployments.

### 4.5 Pitch estimation artifacts in heterogeneous vocalizations

Visual spectrogram inspection revealed systematic discrepancies for subjects S17, S18, S25, and S27: median *f*_0_ estimates fell below 2,300 Hz despite energy concentration above this threshold. This indicates that autocorrelation-based pitch estimators may be tracking residual low-frequency noise near the high-pass filter cutoff (1 kHz) rather than the true vocal fundamental, particularly in noisy, aperiodic, or broadband vocalizations where harmonic structure is ambiguous (Mauch and Dixon, 2014). This artifact explains the weak correlation between estimated *f*_0_ and body size (*R*^2^ = 0.019) despite biomechanical predictions Fitch, (1997).

This measurement error explains two key findings: (1) the 3–8 percentage point improvement when squeal-dominated subjects were excluded, and (2) why *f*_0_ ranks as highly important yet fails as a reliable age predictor. Future work should implement stricter preprocessing (e.g., higher cutoff frequencies or adaptive filtering) and call-type classification as preprocessing (Schneider et al., 2024), extracting pitch parameters only from tonal calls with unambiguous harmonic structure while using alternative spectral descriptors (MFCCs, centroid, bandwidth) for noisy vocalizations.

## 5 Conclusions

Acoustic sex classification from Greater Caribbean manatee vocalizations achieves operationally viable performance (85%–87% accuracy) suitable for passive monitoring, though subject-level bootstrap analysis reveals substantial individual heterogeneity (female 95% CI: 68.7%–96.4%; male: 75.1%–83.6%), indicating 10%–15% of individuals will be systematically misclassified. Operational deployment must incorporate individual-level confidence bounds rather than relying solely on population metrics.

Age classification presents fundamental challenges despite 73%–85% global accuracy, with severe juvenile under-detection (14%–26% recall) and extreme uncertainty (juvenile 95% CI: 9.3%–86.3%, 3.2 × wider than adults). Fundamental frequency shows negligible correlation with body size (*R*^2^ = 0.019), with juvenile and adult *f*_0_ ranges overlapping extensively, preventing discrete boundaries. Threshold optimization improves juvenile detection to 63% but elevates false positives to 37%, representing context-dependent trade-offs for conservation surveillance. Body size estimation via acoustic regression achieved proof-of-concept (MAE = 0.208 m, *R*^2^ = 0.33) supporting coarse profiling into broad categories when integrated with sex classification.

Sex-ratio monitoring provides immediate conservation value for endangered populations in turbid riverine habitats. Data spanning three years across both seasons demonstrate temporal robustness. Age classification requires expanded sampling prioritizing demographic balance (currently *n* = 4 juveniles, 1 female) and longitudinal tracking to characterize vocal ontogeny. Integration of demographic classification with established individual identification frameworks would enable comprehensive acoustic mark-recapture, simultaneously estimating abundance, sex ratios, and demographic structure—complementing ongoing monitoring efforts in the Changuinola River. This study establishes both feasibility and critical limitations of acoustic demographic inference, providing bootstrap-derived uncertainty metrics essential for evidence-based conservation decision-making.

## Data Availability

The raw data supporting the conclusions of this article will be made available by the authors, without undue reservation.
